# Postoperative feeding in neonatal duodenal obstruction

**DOI:** 10.1186/s12887-022-03524-7

**Published:** 2022-08-03

**Authors:** Dolrudee Aroonsaeng, Paul D. Losty, Pornsri Thanachatchairattana

**Affiliations:** 1 Division Of Pediatric Surgery, Department Of Surgery, Faculty Of Medicine Ramathibodi Hospital, Mahidol University, Bangkok, Thailand; 2 Pediatric Surgery Unit, Department of Surgery, Khon Kaen University, Khon Kaen, Thailand; 3 Institute Of Life Course And Medical Sciences, University Of Liverpool, Liverpool, UK

**Keywords:** Duodenal atresia, Duodenal stenosis, Gavage feeding, Gastric reservoir, Oral feeds

## Abstract

**Background:**

Findings from manometry studies and contrast imaging reveal functioning gastric physiology in newborns with duodenal atresia and stenosis. Stomach reservoir function should therefore be valuable in aiding the postoperative phase of gastric feeding. The aim of this study was therefore to compare the feasibility of initiating oral or large volume(s) gavage feeds vs small volume bolus feeds following operation for congenital duodenal anomalies.

**Methods:**

Single-center electronic medical records of all babies with duodenal atresia and stenosis admitted to a university surgical center during January 1997–September 2021 were analyzed. A fast-fed group (FF) included newborns fed with oral or gavage feeds advanced at a rate of at least 2.5 ml/kg and then progressed more than once a day vs slow-fed group (SF) fed with gavage feeds at incremental rate less than 2.5 ml/kg/day for each time period of oral tolerance or by drip feeds. Total feed volume was limited to 120–150 ml/kg/day in the respective study cohort populations.

**Results:**

Fifty-one eligible patients were recruited in the study - twenty-six in FF group and twenty-five in SF group. Statistically significant differences were observed in the (i) date of first oral feeds (POD 7.7 ± 3.2 vs 16.1 ± 7.7: *p* < 0.001), and (ii) first full feeds (POD 12.5 ± 5.3 vs 18.8 ± 9.7: *p* < 0.01) in FF vs SF study groups.

**Conclusion:**

Initial feeding schedules with oral or incremental gavage-fed rates of at least 2.5 ml/kg in stepwise increments and multi-steps per day is wholly feasible in the postoperative feeding regimens of neonates with congenital duodenal disorders. Significant health benefits are thus achievable in these infants allowing an earlier time to acquiring full enteral feeding and their hospital discharge.

## Background

Postoperative feeding in newborns with duodenal atresia and stenosis is often associated with challenges attributed to disordered gastro-duodenal physiology. Atony is considered a major obstacle here for delayed feeding progression and neonates are thus usually fed by slow rate(s) regimens or drip feeds via an orogastric tube. Recent manometry [1] and upper gastrointestinal studies (UGIS) [2] however show that the functioning stomach may be capable physiologically of feeding such newborns after operation for congenital duodenal obstruction. Markedly different observations are noted in other studies detailing patients with jejunoileal atresia were impaired gut motility may be inexorably linked with a disordered enteric nervous system [3, 4].

Most newborns with congenital duodenal atresia and stenosis are term infants and considered at minimal risk for necrotizing enterocolitis (NEC). There may however be theoretical concerns whether perinatal or perioperative stressors could be risk factors for NEC. Rapid feeding or ‘fast track’ regimens could however return the neonate to a more physiologic homeostatic process [5] reducing risks of intestinal failure associated liver disease (IFALD) from prolonged parenteral alimentation [6, 7], with a shortened hospital stay (LOS) and better long-term outcome. In the surgical practice of routine monitoring of newborn gastric residual volumes, some studies have suggested it does not have any clear advantage in accelerating feeding and / or monitoring or the early detection of NEC [1]. Such practices may also be considered to prolong attainment of full enteral feeding [8]. Growing knowledge about the key role(s) of enteral nutrition in stabilizing intestinal integrity and maintaining innate immunity are now well-documented and trophic feeds (10–20 ml/kg/day) may therefore be of great benefit in decreasing risks (%) of postoperative sepsis [9].

Randomized controlled trials (RCT) demonstrate the efficacy and safety of ‘fast feeding’ with advancing rates of some 30–40 ml/kg/day achieved in neonates particularly in an effort to reduce risks of NEC [5, 10]. Gavage-feeds [8] or oral-feeds can thus also be considered in the preterm or very low birthweight infant. We could not find published studies addressing the concepts of initiating ‘fast feeds’, i.e. oral or gavage feeding as described in our current study in the postoperative management of newborns having operation for congenital duodenal disorders although a single study from China has considered ERAS concepts [11]. We therefore undertook a pilot feasibility study to investigate feeding regimens in neonates with duodenal atresia and stenosis.

## Methods

Our study classified patient study groups into fast-fed (FF) and slow-fed (SF) by the method of feeding and incremental rate. FF group included patients fed with oral feed and who were fed with gavage boluses advanced at a rate of at least 2.5 ml/kg and stepped feed increments more than once a day. SF group included those who were fed with a gastric tube drip or who fed with gavage boluses advanced at rates less than 2.5 ml/kg/day.

The daily incremental feeding rate(s) were limited at 30 ml/kg and target volumes of 120–150 ml/kg/day as the standard patient care plan. Decision(s) on starting first feeds were based on the presence / absence of bowel sounds and notable absence of bile or coffee ground gastric content, and its volume(s).

All newborns with an index diagnosis of congenital duodenal atresia or stenosis admitted to the Department of Pediatric Surgery Ramathibodi Hospital Mahidol University Bangkok Thailand during January 1997–September 2021 were enrolled in the study. Electronic medical records detailed the varied classification of the congenital duodenal anomaly, operation(s), feeding regimen(s) and all pertinent demographic data, i.e. gender, birthweight (BW), gestational age (GA) and associated anomalies including chromosomal and cardiac defects.

Gastric content volume 1 day before initiating feeding was considered in the decision algorithm for initiation of feeds. The infants’ ability to tolerate an initial oral test feed was then considered for further fast feeding (FF) as - (i) oral, (ii) combined oral and gavage and (iii) tube feedings. Gastric content volume divided by birth weight (BW) was then calculated. Data analyzed were as follows - (i) postoperative date of starting oral feeds - counted on the day that patient was first fed orally and (ii) the first date of commencement of full feeding counted on the day patients achieved the total required volume per day.

The Faculty Of Medicine Ramathibodi Hospital, Mahidol University Ethics Committee approved the pilot study (No. MURA2020/574).

### Statistical analysis

Data were analyzed with STATA 14 package. Student-t test for continuous variables in normal distribution; Wilcoxon Mann-Whitney test, non-paramedic distribution; Chi-square (χ 2) and Fisher’s exact tests, categorical variables. Pearson correlation estimated the correlation between the studied variables.

## Results

A total of 51 patients were included in the study – (FF: *n* = 26) fast-fed group (FF) and (SF: *n* = 25) slow-fed group (SF). No statistical differences were observed in the demographic data, surgical pathology or associated disorders between the study patient groups, and the operative procedures that were undertaken - Table 1.

**Table 1:** Patients Information

Variables	Total (*n* = 51)	Fast feed (*n* = 26)	Slow feed (*n* = 25)	*P*-value
Gender, n (%)				
Male	28 (54.9)	15 (57.7)	13 (52.0)	0.683
Female	23 (45.1)	11 (42.3)	12 (48.0)	
Birth weight (gm), mean ± SD	2423 ± 524	2554 ± 422	2287 ± 590	0.068
GA (weeks), mean ± SD *n* = 43	36.5 ± 1.8	36.8 ± 1.6	36.3 ± 2.1	0.442
Term, n (%) *n* = 6	6 (11.8)	5 (19.2)	1 (4.0)	0.191
Ward of feed, n (%)				
Ped	35 (68.6)	16 (61.5)	19 (76.0)	0.266
Ped Sx	16 (31.4)	10 (38.5)	6 (24.0)	
Age at surgery (days), median (IQR)	4 (2, 8)	5 (3, 8)	3 (2, 5)	0.117
Procedure, n (%)				
Duodenoduodenostomy	38 (74.5)	18 (69.2)	20 (80.0)	0.493
Duodenojejunostomy	8 (15.7)	5 (19.2)	3 (12.0)	
Web excision	4 (7.8)	3 (11.6)	1 (4.0)	
Duodenoduodenostomy+ Duodenojejunostomy	1 (2.0)	0	1 (4.0)	
Atresia type, n (%)				
Stenosis	14 (27.5)	7 (26.9)	7 (28.0)	0.512
Web	15 (29.4)	10 (38.5)	5 (20.0)	
Cord	5 (9.8)	2 (7.7)	3 (12.0)	
Gap	17 (33.3)	7 (26.9)	10 (40.0)	
Annular pancreas, n (%)				
No	35 (68.6)	20 (76.9)	15 (60.0)	0.193
Yes	16 (31.4)	6 (23.1)	10 (40.0)	
Down’s syndrome, n (%)				
No	30 (58.8)	14 (53.8)	16 (64.0)	0.461
Yes	21 (41.2)	12 (46.2)	9 (36.0)	
Congenital heart disease, n (%)				
No	31 (60.8)	17 (65.4)	14 (56.0)	0.493
Yes	20 (39.2)	9 (34.6)	11 (44.0)	
Others n (%)				
No	31 (60.8)	17 (65.4)	14 (56.0)	0.493
Yes	20 (39.2)	9 (34.6)	11 (44.0)	
Medical disorders n (%)				
No	28 (54.9)	16 (61.5)	12 (48.0)	0.331
Yes	23 (45.1)	10 (38.5)	13 (52.0)	
Complicated surgery, n (%)				
No	37 (72.5)	17 (65.4)	20 (80.0)	0.242
Yes	14 (27.5)	9 (34.6)	5 (20.0)	

Fisher’s exact test > Term, Atresia type, Procedure

Chi-square test > Gender, Ward of feed, Annular pancreas, Down syndrome, Heart disease, Other, Medical problem, Complicated surgery

Wilcoxon Mann-Whitney test (non-normal) > Age at surgery

Student t-test (normal) > Birth weight (gm), GA (weeks)

**Fig. 1:**
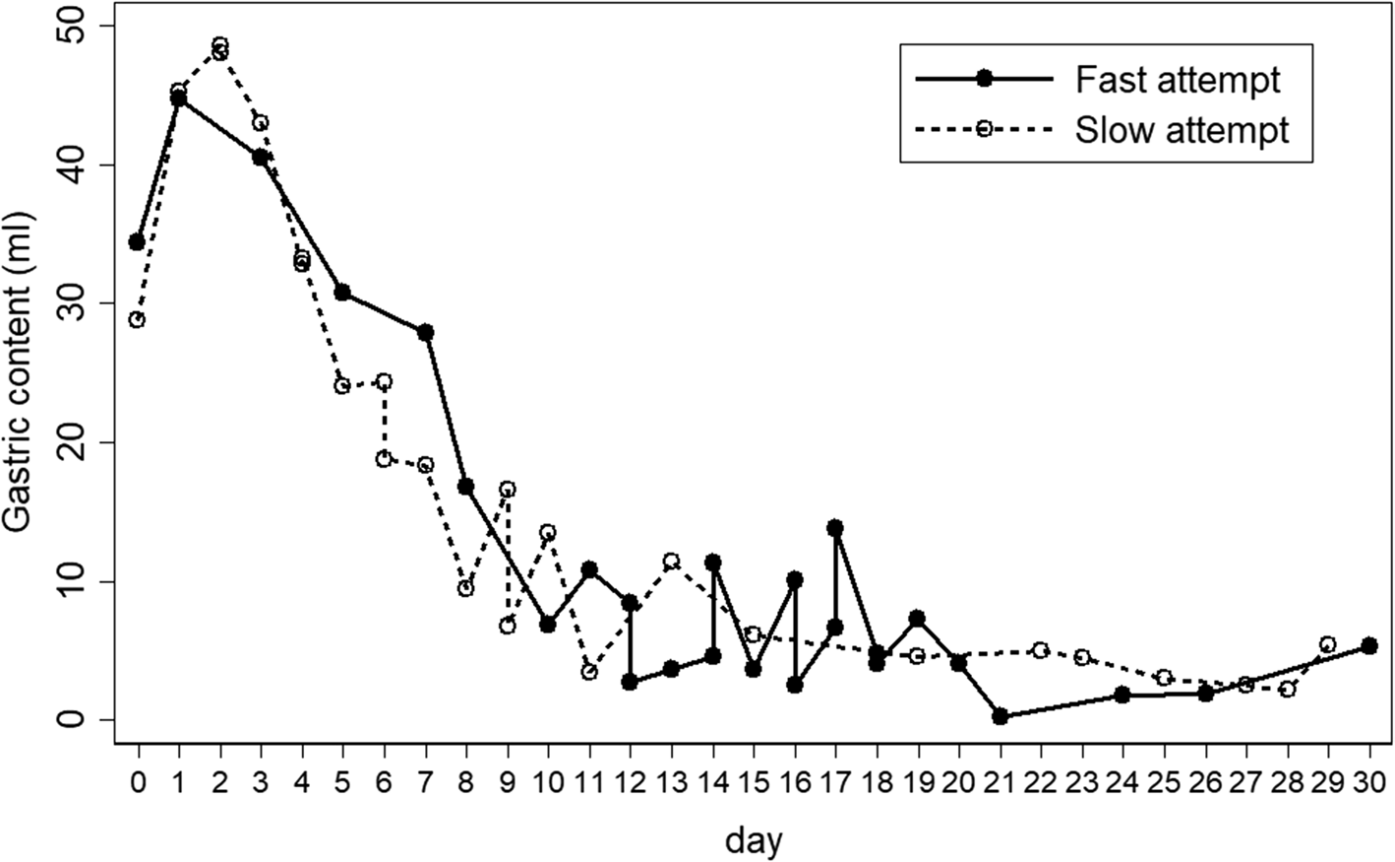
Shows average volume of gastric content (mls) in each postoperative date (days) in patient study groups of fast and slow attempted feeds

**Fig. 2:**
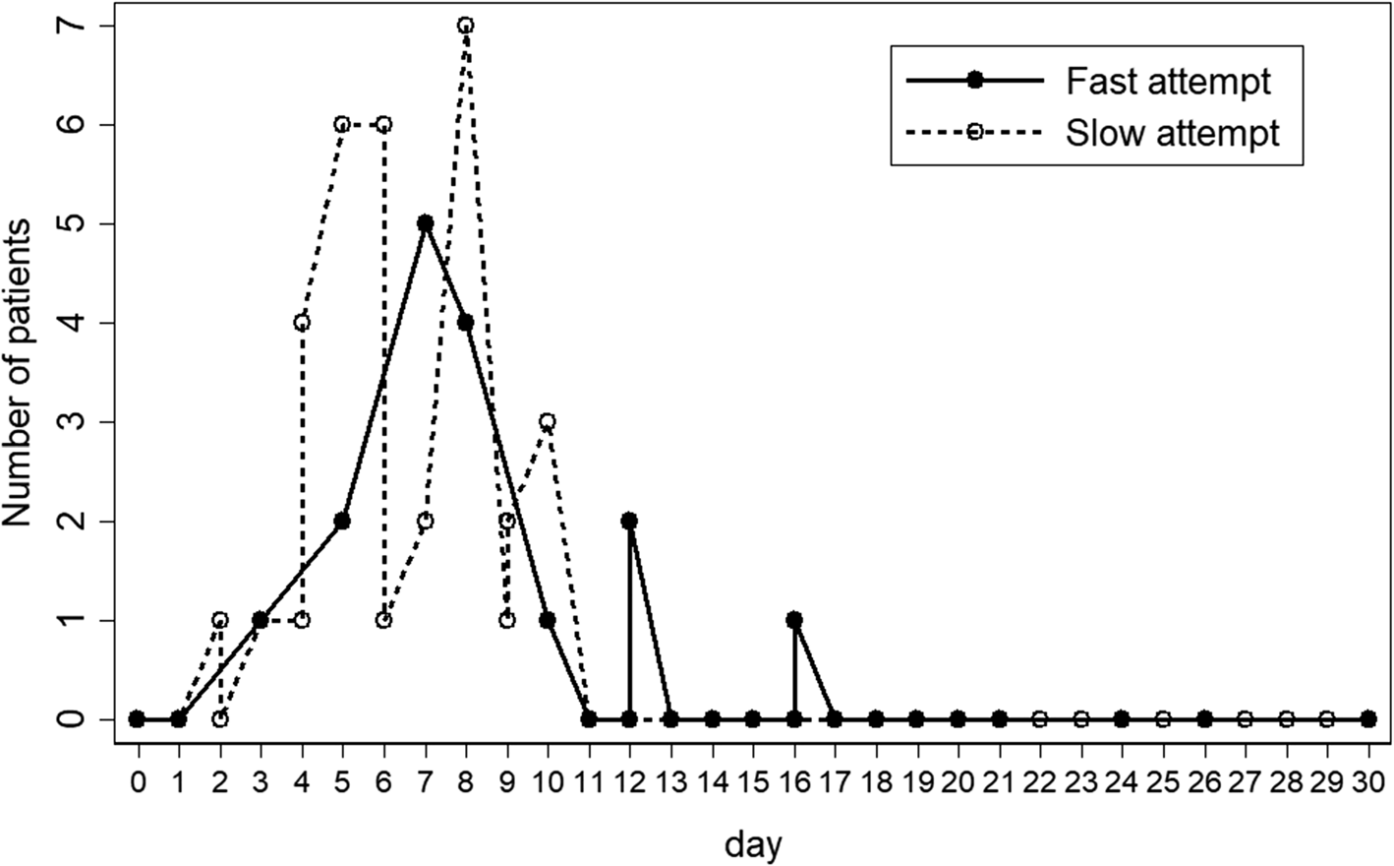
Shows the number of patients in each postoperative date (days) who have commenced type of feeding schedule categorized into Fast and Slow feeds

There were no differences in gestational age(s) of the patient study groups (FF vs SF) GA (mean ± SD: 36.8 ± 1.6, 36.3 ± 2.1, *p* = 0.442) or BW (range: 1510–3500 g, mean ± SD: 2554 ± 422, 2287 ± 590, *p* = 0.068). Pediatricians were responsible for the varied feeding management in FF 61.5% and SF 76% study groups (*p* = 0.266). Median age(s) (IQR) at operation were 5 (3,8) and 3 (2,5) days; *p* = 0.117. Associated disorders considered likely to impact feeding included Down’s syndrome and congenital heart disease. Feeding-associated medical and surgical problems were all not different between the patient study groups (*p* = 0.461, 0.493, 0.331 and 0.242 respectively). Gastric function inferred from the graph is plotted for average gastric content on each postoperative day (Fig. 1) and the number of patients (N) who had first fed on each consecutive postoperative date (Fig. 2). Overall, the patient-studied cohort groups and gastric function were noted to be very similar.

**Fig. 3:**
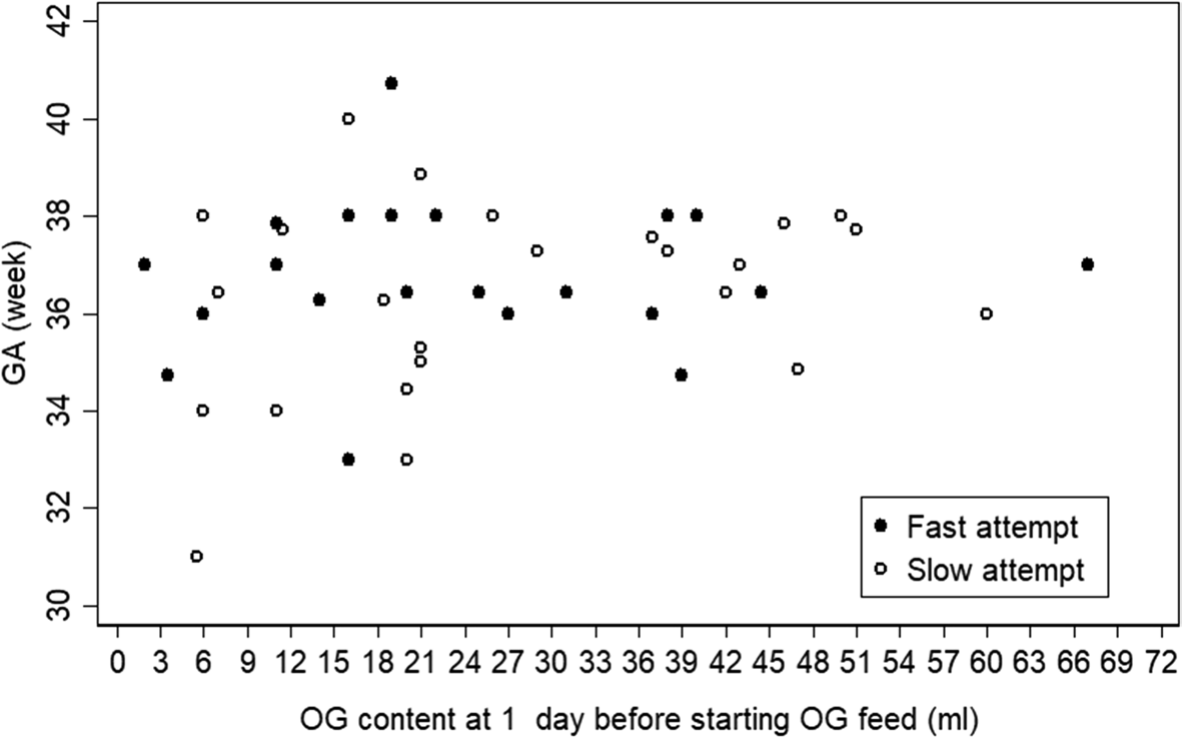
Shows the correlation of GA vs OG content 1 day before feed by feeding type: the overall Correlation (*r* = 0.188), *p*-value = 0.217

**Fig. 4:**
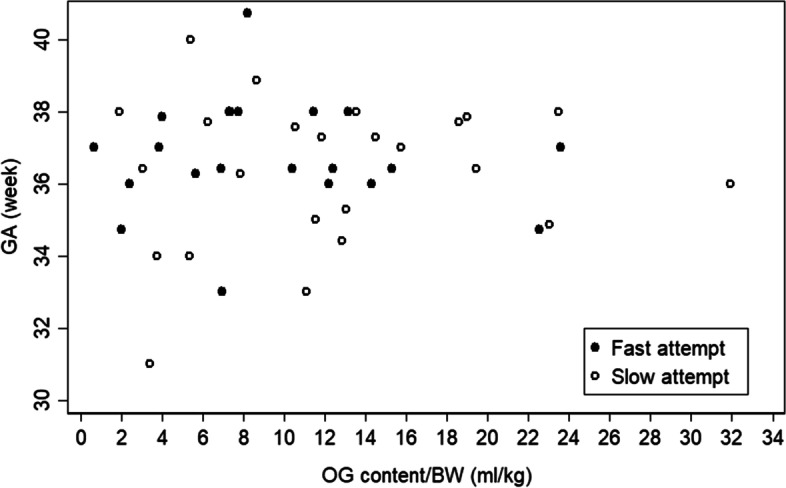
Shows the correlation of GA vs OG content 1 day before feed per BW by feeding type: the overall Correlation (*r* = 0.031), *p*-value = 0.842

**Fig. 5:**
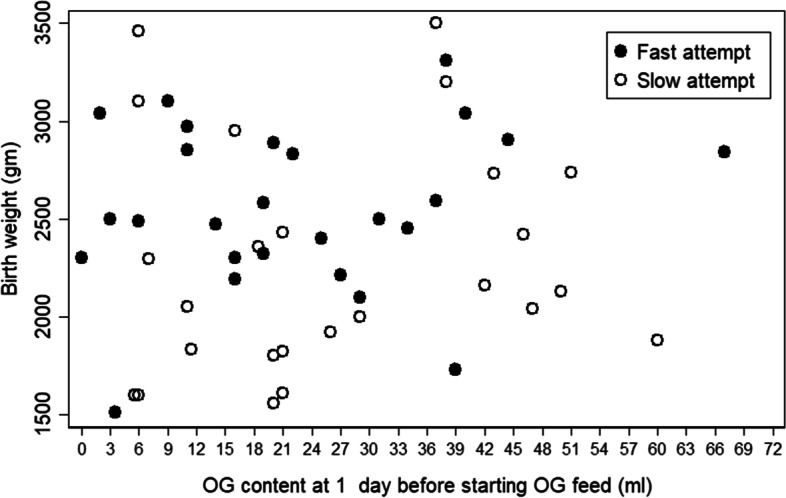
Shows the insignificant correlation (*r* = 0.088, *p* = 0.539) between the volume of gastric content one day before starting feed and birth weight

Table 2 shows that gastric content 1 day before starting feeding was indifferent between study groups (mean ± SD: 22.4 ± 15.9, 26.4 ± 16.7, *p* = 0.391) even when calculated per kilogram of BW (mean ± SD: 8.8 ± 6.3, 11.9 ± 7.6, *p* = 0.125). Analyzing the gastric content 1 day before starting feeding with GA (Fig. 3) and with BW (Fig. 4) showed the correlations (r) 0.188 (*p* = 0.217) and 0.088 (*p* = 0.539), respectively. Moreover, when studying gastric content 1 day before starting feeding per kilogram of BW was correlated to GA (Fig. 5) – (r) was 0.031 (*p* = 0.842) – indicating that the absolute volumes of gastric content before initiating first feeds was much less well correlated to gestational maturity (GA or BW) of the newborns and could therefore not be used as feeding predictor.

**Table 2:** Feeding Data

Variable	Total	Fast feed	Slow feed	*P*-value
OG content at 1 day before starting OG feed (ml), mean ± SD	24.4 ± 16.3	22.4 ± 15.9	26.4 ± 16.7	0.391
OG content at 1 day before starting OG feed /BW (ml/kg), mean ± SD	10.4 ± 7.1	8.8 ± 6.3	11.9 ± 7.6	0.125
First oral feed at POD, mean ± SD	11.9 ± 7.2	7.7 ± 3.2	16.1 ± 7.7	0.000
Full feed at POD, mean ± SD	15.6 ± 8.3	12.5 ± 5.3	18.8 ± 9.7	0.007
Number of dates from start feed to full feed, mean ± SD	8.7 ± 7.2	5.9 ± 4.8	11.5 ± 8.3	0.006
Start feed at POD, mean ± SD	6.9 ± 2.6	6.6 ± 1.7	7.3 ± 3.2	0.314
day2, n (%)	1 (2.0)	0	1 (4.0)	
day3, n (%)	2 (3.9)	1 (3.9)	1 (4.0)	
day4, n (%)	5 (9.8)	1 (3.9)	4 (16.0)	
day5, n (%)	8 (15.7)	6 (23.0)	2 (8.0)	
day6, n (%)	7 (13.7)	6 (23.0)	1 (4.0)	
day7, n (%)	7 (13.7)	2 (7.7)	5 (20.0)	
day8, n (%)	11 (21.6)	7 (26.9)	4 (16.0)	
day9, n (%)	3 (5.9)	2 (7.7)	1 (4.0)	
day10, n (%)	4 (7.8)	1 (3.9)	3 (12.0)	
day12, n (%)	2 (3.9)	0	2 (8.0)	
day16, n (%)	1 (2.0)	0	1 (4.0)	
Max advanced feed volume per day (ml), mean ± SD	7.2 ± 4.5	9.5 ± 4.7	4.9 ± 2.9	0.000
Discharge at POD (day), median (IQR) *n* = 50	18 (13,28)	14 (12, 25)	20 (16, 32)	0.025

Student t-test (normal) > OG content at 1 day before starting OG feed, OG content/BW, start oral feed at POD, full feed at POD for analysis, number of dates from start feed to full feed, Start feed at POD, Max advanced feed volume per day

Wilcoxon Mann-Whitney test (non-normal) > Discharge at POD

The overall increment rate of feeding ranged from 1.92 to 19.56 ml/kg/day; the FF group had on average advanced volume(s) of feed per day more than the SF group (9.5 ± 4.7 vs 4.9 ± 2.9, *p* < 0.001 - Table 2. According to Table 3, the medical pediatricians tended to feed in slowly advanced incremental rates (FF; *n* = 16, SF; *n* = 19 patients) whereas the pediatric surgeons preferred a fast fed-rate (FF; *n* = 10, SF; *n* = 6 patients); *p* = 0.266. Surgeons also preferred feeding newborns in higher daily volumes vs pediatricians (FF: 10.2 ± 4.7 vs 8.9 ± 4.8, *p* = 0.343; SF: 6.2 ± 2.9 vs 4.1 ± 1.7, *p* = 0.175). The practice of gastric content monitoring under whichever clinical service (surgeon vs pediatrician) was designated responsible for the patients’ feeds was statistically insignificant (FF: 20.0% vs 18.8%, *p* = 0.939, SF: 16.7% vs 42.1%, *p* = 0.364). Of particular note in both study cohort groups no single patient developed NEC or other feeding-related complications.

**Table 3:** Categorized details of feed schedules according to pediatrician and surgeons

variable	Ped (*n* = 35)	Ped Sx (*n* = 16)	*P*-value
Fast fed group (*n* = 26)	16	10	
Gastric content monitoring, n (%)			
No	13 (81.3)	8 (80.0)	0.939
Yes	3 (18.8)	2 (20.0)	
Max advanced feed-volume per day (ml), mean + SD	8.9 + 4.8	10.2 + 4.7	0.343
Slow fed group (*n* = 25)	19	6	
Gastric content monitoring, n (%)			
No	11 (57.9)	5 (83.3)	0.364
Yes	8 (42.1)	1 (16.7)	
Max advanced feed-volume per day (ml), mean + SD	4.1 + 1.7	6.2 + 2.9	0.175

Fisher’s exact test > Gastric content monitoring

Student-t test > Max advanced feed-volume per day

Comparing FF and SF groups (Table 2) no differences in the mean postoperative date(s) of first feeding were observed 6.6 ± 1.7 vs 7.3 ± 3.2 (*p* = 0.314). Statistically significant differences were however noted in the first oral-fed date (POD 7.7 ± 3.2 vs 16.1 ± 7.7: *p* < 0.001) and of the first full-fed date (POD 12.5 ± 5.3 vs 18.8 ± 9.7: *p* < 0.01). We also observed marked differences in the number of incremental dates to achieve ‘step-up feeding’ comparing the study groups (5.9 ± 4.8 vs 11.5 ± 8.3: *p* < 0.01). We studied the date(s) from the start of feed to full feeds and discharge dates counted from operative date (i.e. day of operation) instead of hospital stay. The shorter time to hospital discharge were notably significant in the FF study cohort group (14(12,25), 20(16,32): *p* = 0.025).

## Discussion

In this current study, most patients with congenital duodenal disorders were near term and had birth weights > 1500 g. with a considered relatively minimal risk of acquiring NEC. The primary study outcome we sought to test was the ‘feasibility of fast track’ postoperative oral feeding or high-volume gavage feeding in infants with such disorders that would show our hypothesis was applicable with rapid patient recovery and a full return of intestinal gut function in a step-wise physiologic process [5]. The data highlighting residual gastric content on each postoperative date (Fig. 1) we show were comparable and compatible with the study findings of Takahashi and Spilde describing manometry and UGIS [1, 2] illustrating a rapid recovery of gastric physiological function. Combined with gastric reservoir capacity we further show that the practice of early oral feeding or large volume gavage is wholly feasible in shortening patient hospital stay. Patients in both study groups (FF and SF) were similar in terms of demographics and gastric function as revealed in Table 1, Figs. 1 and 2.

To the best of our knowledge there are few (if any) studies addressing the strategy of initiating ‘ oral or gavage feeding ’ fast track concepts in newborns with congenital duodenal disorders as to what may be best considered the ‘ideal method ‘of advancing postoperative feeding [7]. We show that the monitoring of gastric content was of itself less crucial for accelerating feeding [1] thus encouraging the working practice paradigm of ‘scaling up feeds’. After we had assured the recovery of gastrointestinal function with reduction of gastric and bile aspirates, oral feeding or gavage feeds were advanced with daily incremental rates of more than 2.5 ml/kg. Any potential concerns of high-volume postoperative feeding causing foregut anastomotic disruption or NEC were reassuringly not shown in this current study indicating the practical utility of fast-track feeding concepts.

Some interesting studies also report on use of trans-anastomotic (TAT) or trans-pyloric feeding, in congenital duodenal disorders [12–14]. A systematic review and meta-analysis published in 2021; however, revealed insignificant differences in the time(s) taken to achieve full feeds between TAT and non-TAT fed groups showing here also similar outcomes to an earlier study we have previously reported [12, 15].

We herein acknowledge our current report has some limitations as this was a retrospective study with a relatively small number of patients. Moreover, time-related events with patient care may have evolved with varied management strategies deployed during era(s) 1997–2021. The concept(s) of accelerated recovery after surgery for congenital duodenal disorders was commenced by our surgical unit to promote earlier enteral feeding in our clinical practice and we convincingly show it to be of clear benefits in the FF patient-studied cohort group.

## Conclusions

In closing, we have learned that promoting and supporting the initiation of ‘fast track’ oral or advanced gavage-feeds at a rate at least 2.5 ml/kg/increment in multi-steps per day is wholly feasible in the postoperative feeding regimens of newborns with congenital duodenal obstruction. This practice has clear benefits in achieving an earlier time to full enteral feeding and in turn may help to accelerate hospital discharge.

## Data Availability

The datasets used and/or analyzed during the current study are available from the corresponding senior author on reasonable request.

## Abbreviations

FF: Fast fed group
SF: Slow fed group
UGIS: Upper gastrointestinal study
IFALD: Intestinal failure associated liver disease
NEC: Necrotizing enterocolitis
BW: Birthweight
GA: Gestational age at birth
TAT: Trans-anastomotic tube
